# Retention of Habitat Complexity Minimizes Disassembly of Reef Fish Communities following Disturbance: A Large-Scale Natural Experiment

**DOI:** 10.1371/journal.pone.0105384

**Published:** 2014-08-20

**Authors:** Michael J. Emslie, Alistair J. Cheal, Kerryn A. Johns

**Affiliations:** Australian Institute of Marine Science, Townsville, Queensland, Australia; University of Sydney, Australia

## Abstract

High biodiversity ecosystems are commonly associated with complex habitats. Coral reefs are highly diverse ecosystems, but are under increasing pressure from numerous stressors, many of which reduce live coral cover and habitat complexity with concomitant effects on other organisms such as reef fishes. While previous studies have highlighted the importance of habitat complexity in structuring reef fish communities, they employed gradient or meta-analyses which lacked a controlled experimental design over broad spatial scales to explicitly separate the influence of live coral cover from overall habitat complexity. Here a natural experiment using a long term (20 year), spatially extensive (∼115,000 kms^2^) dataset from the Great Barrier Reef revealed the fundamental importance of overall habitat complexity for reef fishes. Reductions of both live coral cover and habitat complexity had substantial impacts on fish communities compared to relatively minor impacts after major reductions in coral cover but not habitat complexity. Where habitat complexity was substantially reduced, species abundances broadly declined and a far greater number of fish species were locally extirpated, including economically important fishes. This resulted in decreased species richness and a loss of diversity within functional groups. Our results suggest that the retention of habitat complexity following disturbances can ameliorate the impacts of coral declines on reef fishes, so preserving their capacity to perform important functional roles essential to reef resilience. These results add to a growing body of evidence about the importance of habitat complexity for reef fishes, and represent the first large-scale examination of this question on the Great Barrier Reef.

## Introduction

Habitat complexity is fundamentally important for the maintenance of high biodiversity across a range of ecosystems [1]–[5]. Coral reef ecosystems are among the most diverse on the planet with reefs with higher habitat complexity often housing more species than less complex reefs due to the greater variety of niches and shelter [6]–[8]. Habitat complexity on coral reefs has two major components; the underlying substrate rugosity and the skeletal structure provided by live and dead hard corals. Coral reefs are subject to many types of disturbance that can have negligible to severe impacts on coral cover and habitat complexity. For example, disturbances such as *Acanthaster planci* (crown-of-thorns starfish) outbreaks and coral bleaching cause coral mortality but leave skeletons intact [9]–[11], so habitat complexity remains largely unchanged in the short term. Subsequently, coral skeletons may erode due to natural processes causing longer term declines in habitat complexity. Conversely, waves from storms can obliterate entire coral colonies removing the habitat complexity previously afforded by their skeletons [11], [12]. However, loss of coral structures due to storms or skeletal erosion will not necessarily lead to low habitat complexity if substrate rugosity is high. Indeed, reefs with high substrate rugosity should maintain a greater diversity of organisms than reefs with low substrate rugosity once hard corals are removed, with the exception of those organisms fundamentally dependent on intact coral skeletons or living coral tissue for survival.

Disturbances on coral reefs can dramatically impact the diversity, abundance and community structure of reef fishes, because many fish species are closely associated with live corals and their structures [6]–[8], [13]–[15]. To date, many studies have attributed changes in fish communities to loss of hard coral cover [9], [13], [16]–[20]. Numerous reef fishes rely on hard corals for food and/or shelter and many of these species decline in abundance following hard coral decline [9], [16]–[22]. However, numerous fish species with seemingly limited reliance on hard corals *per se* (e.g. non-corallivorous butterflyfishes, large predators, some herbivorous fishes) have also declined in abundance following disturbances, and in these cases the role of habitat complexity has been implicated [8], [9], [11], [13], [20], [23]. Declines in abundance and diversity of reef fishes following disturbances can be detrimental to ecosystem functioning and reef resilience due to a reduction in the capacity of reef fishes to perform trophic functions. For example, a reduction in the number and diversity of herbivorous fishes decreases their capacity to prevent proliferation of macro-algae that may limit recovery of corals following disturbances [24]–[32]. Clearly, declines in both live corals and habitat complexity must be important to reef fishes, and disentangling the relative influence of each will provide clues to the relative threat to reef fishes of disturbances which do and do not alter habitat complexity.

It has previously been demonstrated through experimentation [7], [33], [34] and longer term datasets [12]–[14], [16]–[22] that reductions in habitat complexity and live coral cover adversely affect reef fish communities. Manipulative experiments have generally been conducted at restricted spatio- temporal scales, typically small (∼10 s of m^2^) patch reefs surveyed over several months [7], [33], [34], and results are difficult to scale up to ecosystem levels. Projects conducted over larger spatio-temporal scales have generally employed gradient/regression type analyses (e.g. [13]) or meta-analyses (e.g. [11]), which are useful approaches for highlighting relationships among variables, changes in variables along a gradient and for integrating many disparate datasets, but lack rigorous experimental designs with which to definitively attribute causation. Here we use data collected from reefs spread over 115,000 km^2^ of the Great Barrier Reef (GBR), gathered over 20 years and employ a natural experiment to formally test how the loss of live coral versus loss of habitat complexity influences reef fish community structure, the diversity of reef fish families and functional groups, and the abundance of individual species.

## Methods

### Sampling

**Figure 1:**
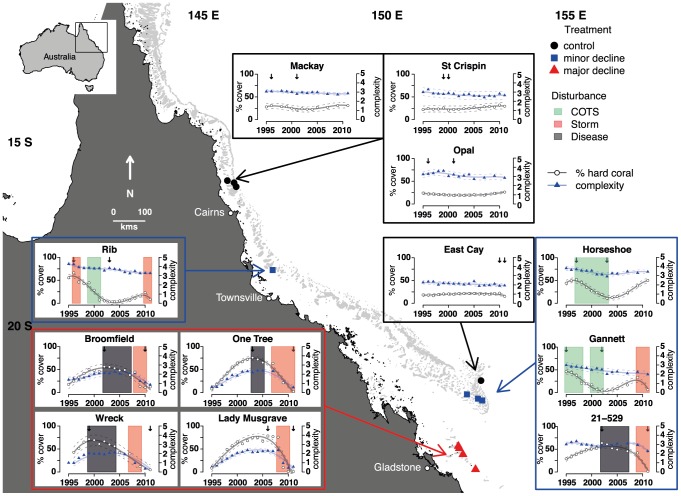
Location of the study reefs in each of the three treatments (Major Decline, Minor Decline and Control). Small panels display trends in hard coral cover and habitat complexity, along with shaded periods of time when disturbances (COTS = *Acanthaster planci* outbreaks, storms & coral disease) occurred. Points are raw data means, while solid lines indicate modelled average trends and dotted lines show 2 x standard errors from a linear mixed effects model fitted separately to hard coral cover and habitat complexity. Arrows mark the years of greatest and least hard coral cover.

Data were gathered as part of the Long Term Monitoring Program at the Australian Institute of Marine Science (GBRMPA permit number G13/36390.1); in which fish and benthic communities have been surveyed on 47 reefs of the GBR since 1995. Large-scale disturbances, such as storms and *A. planci* outbreaks that have occurred over the last two decades on the GBR [22], [35]–[36], facilitate opportunities to test macro-ecological hypotheses that due to their scope, require manipulations of a scale (100 s kilometres) that are logistically impossible for researchers to attempt using traditional experimental frameworks [37].We were able to perform a natural experiment to investigate the effects of reductions in live coral cover versus habitat complexity on reef fish communities, by retrospectively assigning replicate reefs into three treatments based on the effects of disturbances. Eight reefs were chosen based on comparable levels of live coral cover (>50%) and subsequent similar and very large relative declines in cover (∼90%) due to disturbances. These reefs were separated into two equal treatments based on relative reductions in habitat complexity: 1. a major decline in habitat complexity from high/moderate to very low levels (hereafter “Major Decline”), and 2. a minor decline in habitat complexity from high to moderate levels (hereafter “Minor Decline”). A further four reefs had minimal declines in hard coral cover and no change in habitat complexity (hereafter “Control”; Fig. 1). Even though reefs in each treatment were unevenly distributed geographically (Fig. 1), 77% of fish species were common to all reefs in the study thus enabling valid comparisons of changes to fish communities. Furthermore, our analysis determined the magnitude of change in individual species abundance and community structure, plus the proportion of the community affected (irrespective of identity) before and after disturbances. Thus species identity *per se* was not important but rather the magnitude of changes and the proportion of the community affected.

Three sites of five permanently marked 50 m transects were situated in comparable reef slope habitats (n = 15 transects per reef) and were surveyed on SCUBA annually from 1995 until 2006 and then biennially thereafter. From 1995 until 2005, the benthic community was described using a 30-cm video swathe along the transects. Forty frames from each video transect were sampled and the benthic organisms beneath five points projected on to each frame in a quincunx pattern were identified to the finest taxonomic resolution possible, yielding 200 samples per transect. After 2006, a digital still image was taken every metre along each transect, and forty images were selected and analysed as before [38]. These data were then converted to percent cover of total hard coral for use in univariate analyses. For multivariate analyses, data were converted to percent cover of finer taxonomic groupings that included different growth forms of the most abundant coral family Acroporidae and other hard corals (including all other non-Acroporidae hard coral families), fire coral (genus *Millepora*), soft corals, coralline, turf and macro-algae, rubble, dead coral, sand, abiotic, sponges and other (rare benthic organisms of very low abundance e.g., ascidians, anemones). Fish communities were surveyed concurrently on the same transects using underwater visual census. The abundance and number of species of fishes recorded during surveys were taken from a list of 215 mobile, diurnally active species (including the families Acanthuridae, Chaetodontidae, Labridae, Lethrinidae, Lutjanidae, Pomacentridae, Scaridae, Siganidae, Zanclidae and the commercially important *Plectropomus* spp., hereafter “coral trout”). While parrotfishes are now considered as a tribe Scarinae within the family Labridae, we use the term “Scaridae” to distinguish this group of fishes from other Labridae. We define “species richness” as the number of species recorded and use this term hereafter. Cryptic species such as gobies and blennies were not included. Two transect widths were used: 50×1 m belts for the Pomacentridae and 50×5 m belts for the remaining families [39]. Habitat complexity was independently estimated retrospectively by two observers using a scale of zero (least complex - minimal vertical relief, few holes, crevices and overhangs) to five (most complex - high vertical relief, many holes, crevices and overhangs) from 360^o^ video panoramas taken at the start of each transect. This 0 to 5 scale correlates strongly with a range of other rugosity metrics and has been found to be a good predictor of reef fish diversity and abundance [40].

### Analyses

To provide the clearest picture of absolute changes in fish communities under varying degrees of change in habitat complexity, we compared metrics of reef fish communities at times of greatest (hereafter “Before”) and least (hereafter “After”) percent coral cover (indicated by arrows in Fig 1). All analyses were conducted in R [41]. To visualise the changes in fish and benthic communities before and after disturbances, we performed a non-metric Multi-Dimensional Scaling (nMDS) based on the Bray-Curtis similarity co-efficient using the iso-MDS package. To reduce the influence of highly abundant taxa, benthic cover data were row centred and square-root transformed. Similarly, to visualise changes to the whole community rather than a few highly abundant species, fish abundances were row centred and fourth root transformed prior to analysis. To examine the magnitude of change in fish and benthic communities before and after disturbances, we conducted a permutational multivariate analysis of variance using distance matrices and assessed the sums of squares for each Treatment and used the ADONIS function from the VEGAN package in R [41]. As the Treatment by Time interaction was significant, we re-ran the analysis separately for each Treatment (Major Decline, Minor Decline, Control).

Changes in fish and benthic communities were further investigated using Bayesian hierarchical models [42], fitted separately for hard coral cover, habitat complexity, total fish species richness and the species richness of eight reef fish families surveyed (Acanthuridae, Chaetodontidae, Labridae, Lethrinidae, Lutjanidae, Pomacentridae, Scaridae, Siganidae), plus the commercially important coral trout (*Plectropomus* spp.). In order to assess the effects of loss of habitat complexity and live coral on functional roles performed by reef fishes, we examined changes to the species richness of broad functional groups including corallivorous and generalist butterflyfishes, herbivores, planktivores and predators. Models had the fixed factors of Time (Before or After) and Treatment (Major Decline, Minor Decline, Control), and random factors of reef, site and transect. Most variables were modelled against a gaussian distribution in the MCMCglmm package [43]; however some were modelled against negative binomial distributions (log link) to account for zero-inflation and over-dispersion inherent in ecological count data [44] (Table S1). Negative-binomial models were fitted through Just Another Gibbs Sampler (JAGS) via the R2JAGS package in R and used non-informative, flat gaussian priors and the posterior distributions were derived from three Markov chain Monte Carlo (MCMC) (see Table S1 for further model details including number of iterations, burn in and thinning). Model convergence and mixing of Markov chains was assessed visually from trace plots and autocorrelation of the chains was always less than 0.2. Inferences about temporal changes were based on 95% Bayesian Higher Posterior Density (HPD) intervals of cell means predicted from posterior distributions of model parameters. Specific post-hoc contrasts were examined including differences in Time (before and after disturbance) among Treatments and differences among Treatments.

We assessed changes in the abundance of individual reef fish species by plotting a comparable metric to account for differences in initial coral cover [45], calculated as the percent change in abundance from before to after disturbance;
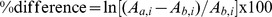
Where A_b_ and A_a_ were mean values at before and after disturbance respectively. Fish species were only included in these analyses if their summed abundance was ≥10 per reef ( = 15 transects) in one of the two years. Changes in individual species abundance were then averaged across the four reefs within each Treatment.

## Results

**Figure 2:**
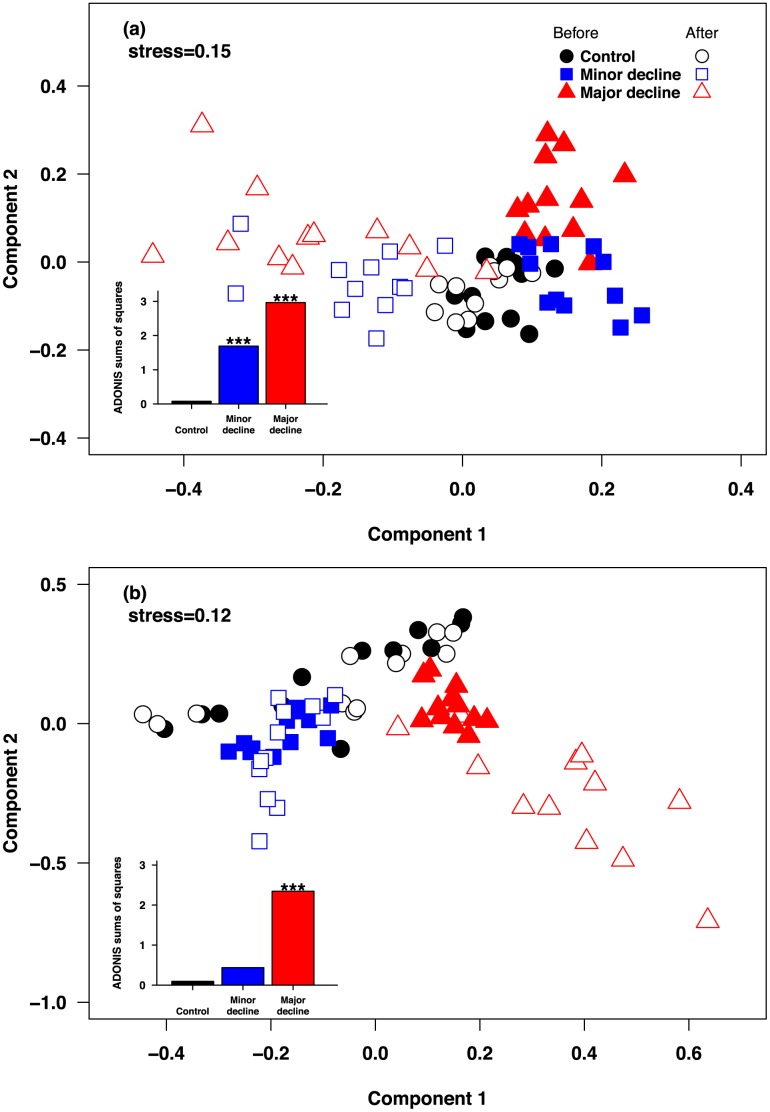
Multi-dimensional plot based on Bray-Curtis similarity coefficients of (a) square-root transformed percent benthic cover and (b) fourth-root transformed fish species abundances. Each panel presents changes to communities following disturbances for the three treatments (Major Decline, Minor Decline and Control). A full model ADONIS analysis revealed a significant interaction for both benthic communities (ADONIS Treatment*Time: F = 14.293, d. f. = 2, Pr(>F) = 0.001) and fish communities (ADONIS Treatment*Time: F = 4.9225, d. f. = 2, Pr(>F) = 0.001). Changes from times of greatest to least coral cover were further examined by separate ADONIS for each individual Treatment (Major Decline, Minor Decline and Control), and the small inset bar graphs display the effect sizes (Sums of Squares) from these individual analyses. ***: Pr(>F) = <0.001

Benthic and fish community structure changed from times of greatest to least coral cover, but the magnitude of change varied among habitat complexity treatments (Fig 2). On reefs with a major decline in complexity, there were substantial shifts in the structure of both fish communities (ADONIS Time: F = 19.134, d. f. = 1, Pr(>F) = 0.001) and benthic communities (ADONIS Time: F = 85.902, d. f. = 1, Pr(>F) = 0.001) (Fig 2). Similarly, a large shift occurred in the benthic communities on reefs with minor declines in habitat complexity, (ADONIS Time: F = 32.429, d. f. = 1, Pr(>F) = 0.001), but a much smaller shift was evident for the fish communities (ADONIS Time: F = 2.1751, d. f. = 1, Pr(>F) = 0.059) on these reefs compared to those in the Major Decline treatment (Fig 2). Very little change occurred in either the fish communities (ADONIS Time: F = 0.3885, d. f. = 1, Pr(>F) = 0.909) or benthic communities (ADONIS Time: F = 1.0507, d. f. = 1, Pr(>F) = 0.304) on Control reefs (Fig 2).

**Figure 3:**
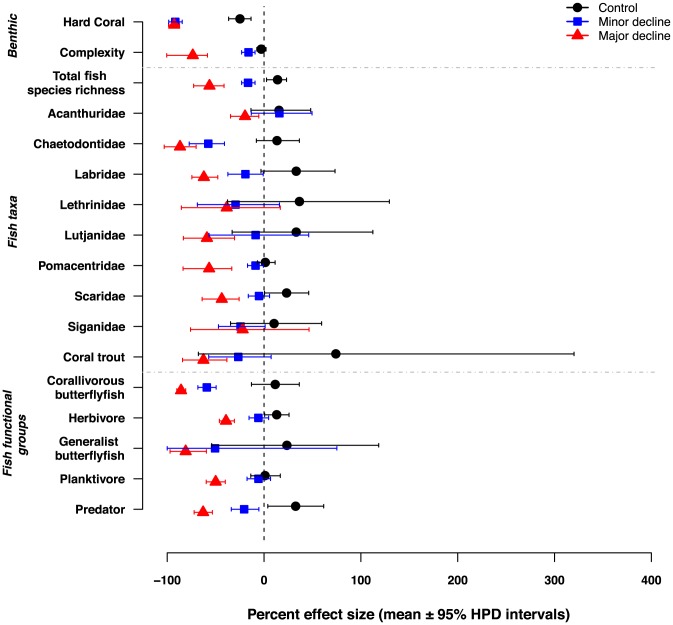
Differences in hard coral cover, habitat complexity, total species richness of fishes and species richness of eight fish families and five broad functional groups for each of the three treatments (Major Decline, Minor Decline and Control). Data are average effect sizes from generalized linear mixed effects model expressed as a per cent change from the time of greatest to least coral cover. Inferences about temporal changes were based on 95% Bayesian Highest Posterior Density (HPD) intervals of cell means predicted from posterior distributions of model parameters derived via Markov-chain Monte Carlo (MCMC) sampling. Effects are considered significant if the HPD intervals do not intersect zero.

Hard coral cover declined in all treatments but the decline was negligible on Control reefs. Habitat complexity only declined substantially on Major Decline reefs; reductions were minimal on reefs in the Minor Decline treatment and were similar to changes at Control reefs (Fig 3). Reductions in fish total species richness and the species richness of the Chaetodontidae and Labridae occurred on reefs in both complexity decline treatments, though the loss was greatest on in the Major Decline reefs (Fig 3). Also, species richness of Acanthuridae, Lutjanidae, Pomacentridae, Scaridae and coral trout declined on reefs in the Major Decline treatment, but not on those in the Minor Decline or Control treatments (Fig 3). There were large declines of species richness of all functional groups of fishes on Major Decline reefs (Fig 3). However, the species richness of only two functional groups, corallivorous butterflyfishes and predators, declined on Minor Decline reefs and these reductions were substantially smaller than those on reefs in the Major Decline treatment. There was no substantial decline in species richness of any functional group on Control reefs (Fig. 3).

**Figure 4:**
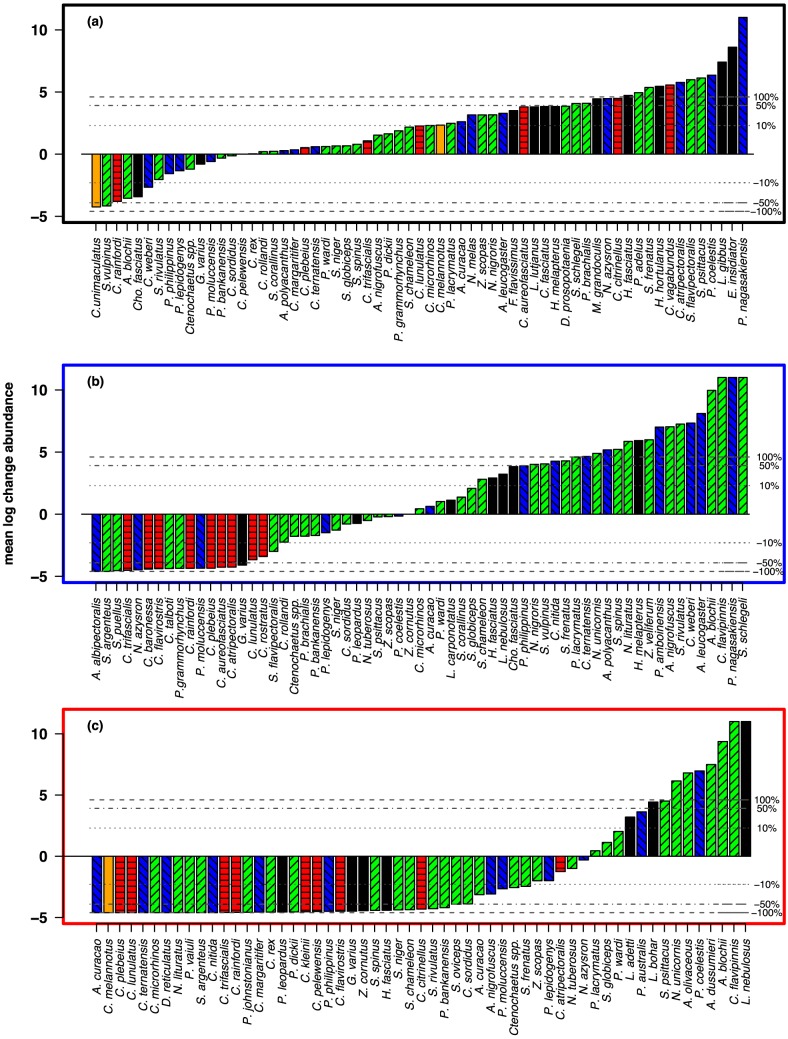
Average percentage change in abundance of individual fish species between times of greatest and least hard coral cover for (a) control reefs (b) reefs that underwent minor declines in complexity (c) reefs that underwent major decline in complexity. Fish species were only included in analyses if their reef wide abundance was ≥10 in one of the two years. Changes in individual species abundance at each reef were then averaged across the four reefs in each Treatment (Major Decline, Minor Decline and Control). Note that the y axis scale is in natural log units and dotted horizontal lines represent 10, 50 and 100% changes in abundance and that error bars were not included to improve clarity. Coloured bars represent trophic affiliations: green with right diagonal hatching = herbivores, blue with left diagonal hatching = planktivores, red with horizontal hatching = corallivorous butterflyfishes, orange solid bars = generalist (non-coral feeding) butterflyfish, black solid bars = predators. A list of species abbreviations on the x-axis and their corresponding species names are found in Table S2.

Changes in the abundance of individual species varied substantially among the three habitat complexity treatments (Fig 4), with major declines in habitat complexity impacting a greater number of species than minor declines. On Major Decline reefs, 75% of species declined in abundance, 56% of species lost half their abundance and 18% were locally extirpated (declined to zero) (Fig 4). In comparison, the abundance of less than half (48%) of the fish species declined on reefs in the Minor Decline treatment, 24% declined in abundance by half and only 3% of species were locally extirpated (Fig 4). Fish species on Control reefs were far less affected; 22% of species declined in abundance, with only 3% declining by half and no species being locally extirpated (Fig 4).

The major loss of habitat complexity also greatly reduced the capacity of reef fishes to perform their functional roles. Among the functionally important herbivorous fishes, fourteen species declined in abundance by 50% or more on reefs that underwent major declines in habitat complexity, compared to four species on reefs with a minor decline and only one species on Control reefs. Additionally, abundances of some commercially important fishery species such as coral trout, were reduced to zero on Major Decline reefs, but declined by less than 5% on Minor Decline reefs. In addition, obligate corallivores accounted for a large proportion of the species that declined in abundance in the Minor Decline treatment, but accounted for a much smaller proportion of the substantially greater number of species that declined on reefs with major declines in complexity.

## Discussion

Using long-term data at ecologically meaningful scales on the GBR, this study has demonstrated the fundamental importance of habitat complexity for the maintenance of diverse fish communities, which is critical for maintaining healthy ecosystem function. Among reefs which underwent large declines in live coral cover, it was only on those reefs where habitat complexity also declined markedly that reef fish communities underwent wholesale reductions in diversity, species abundances and functional capacity. Previously small scale manipulative experiments [7], [33], [34], gradient/regression type analyses [13], [20], or meta-analyses [11] had proposed the importance of habitat complexity for reef fishes, but whether these results reflected a broad-scale truth had not been rigorously tested. Our large-scale, natural experiment was able to demonstrate the generality of habitat complexity as a fundamental driver of reef fish community structure on the GBR, supporting findings in other regions [11], [13], [20], [46], [47]. We showed that major loss of habitat complexity affected a broad array of reef fishes from all trophic/functional groups. Additionally, although major loss of hard coral but not habitat complexity caused declines in some fish species, mostly those intimately associated with hard corals, the role of corals was not as important if overall habitat complexity remained moderate to high. Such results suggest that reefs which undergo major reductions in overall habitat complexity following disturbances will support depauperate reef fish communities, with a reduced ability to perform critical functional roles that contribute to the resilience of coral reefs.

While decreases in abundance of coral dependent species following loss of live coral were expected irrespective of changes in habitat complexity [20], [22], the sweeping reductions in abundance of most reef fish species following major reduction in habitat complexity was more surprising (but see [11], [13]). Large predatory fishes, planktivorous damselfishes and various herbivores were included in these decreases despite most having no obvious dependence on corals, implying that these fishes are dependent on habitat complexity for their survival, most likely through the provision of shelter and food sources. Clearly, habitat complexity affords shelter not only through live corals, but also through dead coral skeletons and by caves, cracks and fissures in the substrate. Where fish abundance declined due to lack of shelter, it was uncertain whether this resulted from migration to more suitable habitat, either around the reef or into deeper water, or from increased mortality resulting from the lack of refugia from predation. Whatever the mechanism of these declines, such dramatic shifts in reef fish community structure have implications for the ecological functioning of coral reef communities.

The extirpation of numerous species of fishes following major declines in habitat complexity contributed to a major reduction in fish diversity, with species from a range of trophic affiliations lost. High fish diversity usually equates to increased functional diversity (the number of functional groups at a site) and functional redundancy (the number of species within a functional group), both key components of reef resilience [48]–[53]. Higher functional diversity should enhance the capacity of a reef to deal with disturbances while functional redundancy provides a form of ecological insurance for the maintenance of a functional role despite losses of some species due to disturbances. Thus it seems highly likely that resilience will be diminished following major losses of habitat complexity. For example, the functional contribution of herbivorous fishes to reef resilience has been well established. Many species of herbivorous reef fishes have the capacity to prevent algal overgrowth and aid coral recovery through their grazing activities, thereby preventing undesirable shifts to a macro-algal dominated state [24], . In this study, the disappearance of fourteen species of herbivorous fishes on reefs where there were major declines of habitat complexity is likely to result in increased vulnerability to such phase shifts (but see [55]).

While the role of herbivorous fishes in reef resilience has been well established, the contributions of many other reef fishes to reef resilience and healthy ecosystem functioning is less clear. However, what is certain is that the loss of a range of coral reef species performing many functional roles will likely have unknown consequences for ecosystem functioning. For example, reductions in the diversity and abundance of corallivorous fishes (e.g. butterflyfishes) will lower coral mortality [56], because corallivorous butterflyfishes can consume between 9 and 13% of the available tissue biomass of coral, representing 50 to 80% of the total annual productivity [57]. The loss of corallivorous fishes following disturbances will therefore remove substantial predation pressures from newly recruited corals and may ultimately aid recovery. Conversely, the loss of corallivorous fishes may deleteriously affect recovery as high diversity and abundance of corallivorous butterflyfishes has been demonstrated to slow or halt the transmission of coral disease [58]. Future research focused on the role played by corallivorous butterflyfishes in coral dynamics shortly following disturbances could aid our understanding of what impact, if any, the loss of corallivorous fishes plays in reef resilience and ecosystem functioning.

It appears that the short term loss following disturbances of adult fishes not directly dependent on live coral relates more closely to the lack of available shelter rather than to loss of living corals *per se*. Similarly, findings of diverse coral reef fish assemblages on artificial structures largely devoid of corals supports the idea that shelter provided by habitat complexity is fundamentally important to coral reef fish communities [59]–[61]. However, many reef fishes use live coral as a cue for settlement, including taxa that do not utilise live coral as adults [18]. Although fish communities may be relatively unaffected by coral mortality when habitat complexity is retained, shifts in community structure may lag behind disturbances if fish recruitment is suppressed by limited availability of living coral, while natural mortality of surviving fishes continues. Furthermore, the erosion of coral skeletons after some disturbances such as *A. planci* outbreaks, coral bleaching and coral disease slowly decreases habitat complexity, and may also produce lagged declines in fishes [20], [62]. However, in this study adult fish populations were not depleted while habitat complexity remained, providing a buffer to fish population declines while coral is recovering in those cases. Thus in normal circumstances, lagged effects are likely to be balanced by coral recovery and new fish recruitment as long as complexity remains following disturbance. Nevertheless, lagged effects in reef fishes may potentially become more important in future decades, especially if predictions of increased coral bleaching and ocean acidification are correct [63]. In summary, while the retention of habitat complexity reduces the short term impact of disturbances on fish communities, the regeneration of live coral is essential for the maintenance of complex habitats and therefore, to the recovery and long term persistence of diverse reef fish communities.

While previous studies have identified the link between habitat complexity and reef fishes, many of these studies have focused on subsets of the fish community (e.g. [20], [21], [64], but see [46]). We were able to tease apart the roles of reductions in coral cover versus habitat complexity on a large proportion of diurnally active and conspicuous reef fish communities over ecologically meaningful scales. To our knowledge, this is the first large-scale natural experiment conducted on the GBR to investigate the fundamental contribution of habitat complexity in driving reef fish community change. These results illustrated that reef fish communities are more adversely affected by disturbances which degrade both live coral cover and habitat complexity (i.e. storms), than those which reduce cover of live corals only (i.e. coral bleaching and outbreaks of *A. planci*). Such results should be of interest to reef managers, particularly given our finding that the major fishery target species, the coral trout (*Plectropomus* spp.) disappeared from sites of major complexity decline, with socio-economic ramifications for fishers utilising this resource. In addition, the impact of storms on reef fish communities at sites where coral skeletons account for most of the habitat complexity will be equally devastating irrespective of any zoning to protect target species from fishing. In effect, the benefits afforded by reserve zoning can be reversed almost instantaneously. Conversely, protection of fish communities at sites where complexity of the underlying substrate is high would better preserve important functional processes performed by reef fishes, encouraging rapid recovery in the event that coral cover is removed. Given the prospect of increases in storm intensity with climate change [65] which may lead to the architectural collapse of coral reefs [66], protecting sites with high underlying substrate complexity should be considered to alleviate vulnerability to disassembly of reef fish communities, reductions in the functional roles they perform and much diminished reef resilience.

## Supporting Information

Table S1 **Model specification for hard coral cover, complexity, total species richness and the species richness of eight families of reef fishes.**
(DOCX)

Table S2 **Full names for species codes and trophic affiliations used in** **Figure 4** **.**
(DOCX)
