# Supplementary material for: Retention of Habitat Complexity Minimizes Disassembly of Reef Fish Communities following Disturbance: A Large-Scale Natural Experiment
Source: PLoS One. 2014 Aug 20;9(8):e105384. doi: 10.1371/journal.pone.0105384 (PMC4139330; doi:10.1371/journal.pone.0105384)
Supplement: Table S1 — Model specification for hard coral cover, complexity, total species richness and the species richness of eight families of reef fishes. (DOCX) [file pone.0105384.s001.docx]

|  | Variable | Zero Inflation | Model  Distribution | Package | Iterations | Burn in | Effective samples |
| --- | --- | --- | --- | --- | --- | --- | --- |
| Benthic | Hard coral cover | No | Gaussian | MCMCglmm | 50,000 | 10,000 | 2,000 |
|  | Habitat complexity | No | Gaussian | MCMCglmm | 50,000 | 10,000 | 3,497 |
| Fish  species richness | Total | No | Gaussian | MCMCglmm | 50,000 | 10,000 | 2,000 |
|  | Acanthuridae | No | Gaussian | MCMCglmm | 50,000 | 10,000 | 2,431 |
|  | Chaetodontidae | No | Gaussian | MCMCglmm | 50,000 | 10,000 | 2,000 |
|  | Labridae | Yes | Negative binomial | JAGS | 1,000,000 | 100,000 | 5,400 |
|  | Lethrinidae | Yes | Negative binomial | JAGS | 1,000,000 | 100,000 | 5,900 |
|  | Lutjanidae | Yes | Negative binomial | JAGS | 1,000,000 | 100,000 | 5,900 |
|  | Pomacentridae | No | Gaussian | MCMCglmm | 50,000 | 10,000 | 2,000 |
|  | Scaridae | No | Gaussian | MCMCglmm | 50,000 | 10,000 | 2,317 |
|  | Siganidae | Yes | Negative binomial | JAGS | 1,000,000 | 100,000 | 5,400 |
|  | Coral Trout | Yes | Negative binomial | JAGS | 1,000,000 | 100,000 | 5,400 |
| Fish trophic species richness | Corallivorous butterflyfish | Yes | Negative binomial | JAGS | 1,000,000 | 100,000 | 5,900 |
|  | Herbivores | Yes | Negative binomial | JAGS | 1,000,000 | 100,000 | 2,700 |
|  | Generalist butterflyfish | Yes | Negative binomial | JAGS | 1,000,000 | 100,000 | 1,700 |
|  | Planktivores | Yes | Negative binomial | JAGS | 1,000,000 | 100,000 | 5,900 |
|  | Predators | Yes | Negative binomial | JAGS | 1,000,000 | 100,000 | 770 |
