# Supplementary material for: Retention of Habitat Complexity Minimizes Disassembly of Reef Fish Communities following Disturbance: A Large-Scale Natural Experiment
Source: PLoS One. 2014 Aug 20;9(8):e105384. doi: 10.1371/journal.pone.0105384 (PMC4139330; doi:10.1371/journal.pone.0105384)
Supplement: Table S2 — Full names for species codes and trophic affiliations used in Figure 4 . (DOCX) [file pone.0105384.s002.docx]

| **Figure abbreviation** | **Name** | **Family** | **Trophic group** |
| --- | --- | --- | --- |
| *A. polyacanthus* | *Acanthochromis polyacanthus* | Pomacentridae | Planktivore |
| *A. albipectoralis* | *Acanthurus albipectoralis* | Acanthuridae | Planktivore |
| *A. blochii* | *Acanthurus blochii* | Acanthuridae | Herbivore |
| *A. dussumeiri* | *Acanthurus dussumieri* | Acanthuridae | Herbivore |
| *A. nigrofuscus* | *Acanthurus nigrofuscus* | Acanthuridae | Herbivore |
| *A. olivaceous* | *Acanthurus olivaceus* | Acanthuridae | Herbivore |
| *A. curacao* | *Amblyglyphidodon curacao* | Pomacentridae | Planktivore |
| *A. leucogaster* | *Amblyglyphidodon leucogaster* | Pomacentridae | Planktivore |
| *C. aureofasciatus* | *Chaetodon aureofasciatus* | Chaetodontidae | Corallivorous butterflyfish |
| *C. baronessa* | *Chaetodon baronessa* | Chaetodontidae | Corallivorous butterflyfish |
| *C. citrinellus* | *Chaetodon citrinellus* | Chaetodontidae | Corallivorous butterflyfish |
| *C. flavirostris* | *Chaetodon flavirostris* | Chaetodontidae | Corallivorous butterflyfish |
| *C. kleinii* | *Chaetodon kleinii* | Chaetodontidae | Corallivorous butterflyfish |
| *C. lunulatus* | *Chaetodon lunulatus* | Chaetodontidae | Corallivorous butterflyfish |
| *C. melonnotus* | *Chaetodon melannotus* | Chaetodontidae | Generalist butterflyfish |
| *C. pelewensis* | *Chaetodon pelewensis* | Chaetodontidae | Corallivorous butterflyfish |
| *C. plebeius* | *Chaetodon plebeius* | Chaetodontidae | Corallivorous butterflyfish |
| *C. rainfordi* | *Chaetodon rainfordi* | Chaetodontidae | Corallivorous butterflyfish |
| *C. trifascialis* | *Chaetodon trifascialis* | Chaetodontidae | Corallivorous butterflyfish |
| *C. unimaculatus* | *Chaetodon unimaculatus* | Chaetodontidae | Generalist butterflyfish |
| *C. vagabundus* | *Chaetodon vagabundus* | Chaetodontidae | Corallivorous butterflyfish |
| *C. fasciatus* | *Cheilinus fasciatus* | Labridae | Predator |
| *C. rostratus* | *Chelmon rostratus* | Chaetodontidae | Corallivorous butterflyfish |
| *C. microrhinos* | *Chlorurus microrhinos* | Scaridae | Herbivore |
| *C. sordidus* | *Chlorurus sordidus* | Scaridae | Herbivore |
| *Cho. fasciatus* | *Choerodon fasciatus* | Labridae | Predator |
| *C. atripectoralis* | *Chromis atripectoralis* | Pomacentridae | Planktivore |
| *C. margaritifer* | *Chromis margaritifer* | Pomacentridae | Planktivore |
| *C. nitida* | *Chromis nitida* | Pomacentridae | Planktivore |
| *C. ternatensis* | *Chromis ternatensis* | Pomacentridae | Planktivore |
| *C. weberi* | *Chromis weberi* | Pomacentridae | Planktivore |
| *C. flavipinnis* | *Chrysiptera flavipinnis* | Pomacentridae | Herbivore |
| *C.rex* | *Chrysiptera rex* | Pomacentridae | Planktivore |
| *C. rollandi* | *Chrysiptera rollandi* | Pomacentridae | Herbivore |
| *C. talboti* | *Chrysiptera talboti* | Pomacentridae | Herbivore |
| *Ctenochaetus spp.* | *Ctenochaetus spp.* | Acanthuridae | Herbivore |
| *D. reticulatus* | *Dascyllus reticulatus* | Pomacentridae | Planktivore |
| *D. prosopotaenia* | *Dischistodus prosopotaenia* | Pomacentridae | Herbivore |
| *E. insidiator* | *Epibulus insidiator* | Labridae | Predator |
| *F. flavissimus* | *Forcipiger flavissimus* | Chaetodontidae | Predator |
| *G. varius* | *Gomphosus varius* | Labridae | Predator |
| *H. hortulanus* | *Halichoeres hortulanus* | Labridae | Predator |
| *H. fasciatus* | *Hemigymnus fasciatus* | Labridae | Predator |
| *H. melapterus* | *Hemigymnus melapterus* | Labridae | Predator |
| *L. nebulosus* | *Lethrinus nebulosus* | Lethrinidae | Predator |
| *L. adetti* | *Lutjanus adetti* | Lutjanidae | Predator |
| *L. bohar* | *Lutjanus bohar* | Lutjanidae | Predator |
| *L. carponotatus* | *Lutjanus carponotatus* | Lutjanidae | Predator |
| *L. gibbus* | *Lutjanus gibbus* | Lutjanidae | Predator |
| *L. lutjanus* | *Lutjanus lutjanus* | Lutjanidae | Predator |
| *M. grandoculis* | *Monotaxis grandoculis* | Lethrinidae | Predator |
| *N. lituratus* | *Naso lituratus* | Acanthuridae | Herbivore |
| *N. tuberosus* | *Naso tuberosus* | Acanthuridae | Herbivore |
| *N. unicornis* | *Naso unicornis* | Acanthuridae | Herbivore |
| *N. melas* | *Neoglyphidodon melas* | Pomacentridae | Planktivore |
| *N. nigroris* | *Neoglyphidodon nigroris* | Pomacentridae | Herbivore |
| *N. azysron* | *Neopomacentrus azysron* | Pomacentridae | Planktivore |
| *P. dickii* | *Plectroglyphidodon dickii* | Pomacentridae | Herbivore |
| *P. johnstonianus* | *Plectroglyphidodon johnstonianus* | Pomacentridae | Herbivore |
| *P. lacrymatus* | *Plectroglyphidodon lacrymatus* | Pomacentridae | Herbivore |
| *P. leopardus* | *Plectropomus leopardus* | Serranidae | Predator |
| *P. adelus* | *Pomacentrus adelus* | Pomacentridae | Herbivore |
| *P. amboinensis* | *Pomacentrus amboinensis* | Pomacentridae | Planktivore |
| *P. australis* | *Pomacentrus australis* | Pomacentridae | Planktivore |
| *P. bankanensis* | *Pomacentrus bankanensis* | Pomacentridae | Herbivore |
| *P. brachialis* | *Pomacentrus brachialis* | Pomacentridae | Herbivore |
| *P. coelestis* | *Pomacentrus coelestis* | Pomacentridae | Planktivore |
| *P. grammorhynchus* | *Pomacentrus grammorhynchus* | Pomacentridae | Herbivore |
| *P. lepidogenys* | *Pomacentrus lepidogenys* | Pomacentridae | Planktivore |
| *P. moluccensis* | *Pomacentrus moluccensis* | Pomacentridae | Planktivore |
| *P. nagasakiensis* | *Pomacentrus nagasakiensis* | Pomacentridae | Planktivore |
| *P. philippinus* | *Pomacentrus philippinus* | Pomacentridae | Planktivore |
| *P. vaiuli* | *Pomacentrus vaiuli* | Pomacentridae | Herbivore |
| *P. wardi* | *Pomacentrus wardi* | Pomacentridae | Herbivore |
| *S. chameleon* | *Scarus chameleon* | Scaridae | Herbivore |
| *S. flavipectoralis* | *Scarus flavipectoralis* | Scaridae | Herbivore |
| *S. frenatus* | *Scarus frenatus* | Scaridae | Herbivore |
| *S. globiceps* | *Scarus globiceps* | Scaridae | Herbivore |
| *S. niger* | *Scarus niger* | Scaridae | Herbivore |
| *S. oviceps* | *Scarus oviceps* | Scaridae | Herbivore |
| *S. psittacus* | *Scarus psittacus* | Scaridae | Herbivore |
| *S. rivulatus* | *Scarus rivulatus* | Scaridae | Herbivore |
| *S. schlegeli* | *Scarus schlegeli* | Scaridae | Herbivore |
| *S. spinus* | *Scarus spinus* | Scaridae | Herbivore |
| *S. argenteus* | *Siganus argenteus* | Siganidae | Herbivore |
| *S. corallines* | *Siganus corallinus* | Siganidae | Herbivore |
| *S. puellus* | *Siganus puellus* | Siganidae | Herbivore |
| *S. vulpinus* | *Siganus vulpinus* | Siganidae | Herbivore |
| *Z. cornutus* | *Zanclus cornutus* | Zanclidae | Predator |
| *Z. scopas* | *Zebrasoma scopas* | Acanthuridae | Herbivore |
| *Z. veliferum* | *Zebrasoma veliferum* | Acanthuridae | Herbivore |
